# The Use of Ultra-Fast Gas Chromatography for Fingerprinting-Based Classification of Zweigelt and Rondo Wines with Regard to Grape Variety and Type of Malolactic Fermentation Combined with Greenness and Practicality Assessment

**DOI:** 10.3390/molecules29194667

**Published:** 2024-10-01

**Authors:** Anna Stój, Wojciech Wojnowski, Justyna Płotka-Wasylka, Tomasz Czernecki, Ireneusz Tomasz Kapusta

**Affiliations:** 1 Department of Biotechnology, Microbiology and Human Nutrition, Faculty of Food Science and Biotechnology, University of Life Sciences in Lublin, 8 Skromna Street, 20-704 Lublin, Poland; tomasz.czernecki@up.lublin.pl; 2 Department of Analytical Chemistry, Faculty of Chemistry, Gdańsk University of Technology, 11/12G Narutowicza Street, 80-233 Gdańsk, Poland; wojciech.wojnowski@pg.edu.pl (W.W.); juswasyl@pg.edu.pl (J.P.-W.); 3 Department of Chemistry, University of Oslo, 0315 Oslo, Norway; 4 Department of Food Technology and Human Nutrition, College of Natural Sciences, University of Rzeszow, 4 Zelwerowicza Street, 35-601 Rzeszów, Poland; ikapusta@ur.edu.pl

**Keywords:** ultra-fast GC, varietal classification, malolactic fermentation, machine learning, AGREE, BAGI

## Abstract

In food authentication, it is important to compare different analytical procedures and select the best method. The aim of this study was to determine the fingerprints of Zweigelt and Rondo wines through headspace analysis using ultra-fast gas chromatography (ultra-fast GC) and to compare the effectiveness of this approach at classifying wines based on grape variety and type of malolactic fermentation (MLF) as well as its greenness and practicality with three other chromatographic methods such as headspace solid-phase microextraction/gas chromatography-mass spectrometry with carboxen-polydimethylosiloxane fiber (SPME/GC-MS with CAR/PDMS fiber), headspace solid-phase microextraction/gas chromatography-mass spectrometry with polyacrylate fiber (SPME/GC-MS with PA fiber), and ultra performance liquid chromatography–photodiode array detector-tandem mass spectrometry (UPLC-PDA-MS/MS). Principal Component Analysis (PCA) revealed that fingerprints obtained using all four chromatographic methods were suitable for classification using machine learning (ML). Random Forest (RF) and Support Vector Machines (SVM) yielded accuracies of at least 99% in the varietal classification of Zweigelt and Rondo wines and therefore proved suitable for robust fingerprinting-based Quality Assurance/Quality Control (QA/QC) procedures. In the case of wine classification by the type of MLF, the classifiers performed slightly worse, with the poorest accuracy of 91% for SVM and SPME/GC-MS with CAR/PDMS fiber, and no less than 93% for the other methods. Ultra-fast GC is the greenest and UPLC-PDA-MS/MS is the most practical of the four chromatographic methods.

## 1. Introduction

The winemaking process for red wines consists of two fermentations: alcoholic fermentation (AF) carried out by yeast and malolactic fermentation (MLF) carried out by lactic acid bacteria (LAB). MLF causes the deacidification and microbiological stabilization of wine as well as producing organoleptic changes in wine that affect its final quality. Both fermentations can occur spontaneously through the activity of the yeast and bacteria naturally occurring in must and wine (spontaneous fermentation), or by inoculation with commercially available yeast and LAB starter cultures (induced fermentation) [1]. Spontaneous MLF is highly unpredictable and may lead to microbiological problems associated with delayed MLF, an increase in volatile acidity, and the formation of undesirable biogenic amines. By contrast, induced MLF reduces the risk of spoilage by other LAB (other than *Oenococcus oeni*) and ensures the rapid onset of MLF and better control over the production of flavor and aroma compounds, as well as preventing the accumulation of biogenic amines [2,3]. To our knowledge, no methodology has been developed to date to rapidly assess the type of MLF applied in the winemaking process.

Wine is a food product that is easily adulterated due to its wide availability around the world and chemical properties such as low pH and high alcohol content [4]. It is impossible to quantify the number of counterfeit wines, but it has been estimated that their share in the global market is $3 billion per year. Wine adulterations result in a loss of revenue for wineries at the same time undermining consumer confidence in the authenticity of premium wines. Counterfeit wines may also contain dangerous chemicals such as methanol, which can cause complications for human health [5]. Wine adulteration may involve the admixture of water, sugar, glycerin, and dyes, as well as false declarations regarding grape variety, geographical origin, or vintage [6,7]. Wine authentication is the verification of a product’s identity to ensure that it conforms to the label declaration [8], e.g., with respect to variety. Grape varieties differ in color, size, shape, and chemical composition. Grape color, skin thickness, and contents of sugars, acids, volatiles, and polyphenols impact the color, aroma, and flavor of wines, thus differentiating wines produced from different grape varieties [9].

Wine authentication is necessary to protect consumers from unfair practices. The varietal authentication or classification of wines is carried out using chromatographic, spectrometric, spectroscopic, and molecular biology methods [7]. Chromatographic methods include high-performance liquid chromatography-photodiode array (HPLC-PDA) detection, liquid chromatography-tandem mass spectrometry (LC-MS/MS), and ultra performance liquid chromatography–photodiode array detector-tandem mass spectrometry (UPLC-PDA-MS/MS) for the determination of phenolic compounds, and gas chromatography-mass spectrometry (GC-MS) and comprehensive two-dimensional gas chromatography mass spectrometry (GCxGC/MS) for the determination of volatile compounds [10,11,12,13,14,15]. The above-mentioned chromatographic methods are usually the most sensitive and specific but require the use of expensive equipment and skilled operators. Moreover, they are costly as well as labor- and time-consuming because they involve multiple steps, such as sample preparation, separation, and identification of compounds. In addition, they may involve the use of toxic reagents and solvents. These problems can be eliminated by using ultra-fast gas chromatography (ultra-fast GC), a method that enables the direct, quick, and effective determination of the varietal authenticity of wines. Ultra-fast GC requires practically no sample preparation, which reduces analysis time and eliminates the need for reagents. While the method’s applicability for the comprehensive determination of volatiles in complex mixtures such as the headspace of a wine sample is limited due to the short length of chromatographic columns and steep temperature ramps, it reliably produces profiles of volatile compounds, called fingerprints, that can be used to rapidly discriminate between different samples [9,16,17].

Ultra-fast GC sits well with the modern approach to analytical chemistry, since it aligns with the principles of Green Analytical Chemistry (GAC) and White Analytical Chemistry (WAC). GAC focuses on green aspects of analytical methods including the safety of solvents and reagents, the formation of toxic waste, energy consumption, and the safety of the analysts [18,19,20,21,22]. WAC is an extension and complement to GAC as it covers the analytical, ecological, and practical aspects of analytical procedures [20]. A number of metric tools for the evaluation of the greenness of analytical methodologies/approaches (e.g., conventional, state-of-the-art, newly developed) have been created and implemented in recent publications: the National Environmental Method Index (NEMI), analytical eco-scale, green analytical procedure index (GAPI), Analytical Greenness application (AGREE), complementary green analytical procedure index (ComplexGAPI), and analytical greenness metric for sample preparation (AGREEprep) [20,23]. The AGREE calculator, which was used in this present study, has 12 assessment criteria taken from the 12 GAC principles transformed into a unified 0−1 scale. Each criterion can be assigned different weights. The final result is the product of the assessment scores for each criterion. The overall score and the corresponding color are shown in the middle of a clock-like graph. The performance of the method in each criterion is reflected with a red–yellow–green color scale, while the weight of each criterion is reflected with the width of its corresponding segment [23]. For an estimation of the practicality of analytical methods, the Red–Green–Blue (RGB) model and Blue Applicability Grade Index (BAGI) have been proposed [23,24]. BAGI evaluates the practicality of an analytical method. It generates a blue asteroid pictogram together with the respective score, which indicates the practicality of a procedure across ten attributes. The color blue has been inspired by the corresponding component of the RGB model [20].

In Poland, there have been cases of mislabelling with a false declaration of grape variety, e.g., designating a Rondo wine as a Zweigelt wine to obtain a higher profit. Rondo is one of the most frequently cultivated red grape varieties, while Zweigelt is much less common [13,14]. The aim of the present study was two-fold: firstly to determine volatile compound fingerprints of Zweigelt and Rondo wines using ultra-fast GC and secondly to compare the effectiveness of this method with regard to the varietal classification of wines and assess the type of MLF, as well as the greenness and blueness of this approach with three other chromatographic methods described in our previous publications: UPLC-PDA-MS/MS, headspace solid-phase microextraction/gas chromatography-mass spectrometry with carboxen-polydimethylosiloxane fiber (SPME/GC-MS with CAR/PDMS fiber), and headspace solid-phase microextraction/gas chromatography-mass spectrometry with polyacrylate fiber (SPME/GC-MS with PA fiber) [13,14,25].

## 2. Results and Discussion

**Figure 1:**
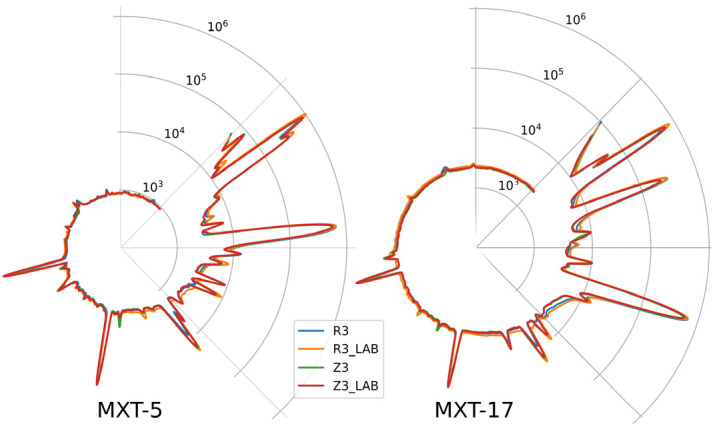
Radar plots of the chromatograms of selected wines obtained with ultra-fast GC; the circumference of the plots denotes retention time (100 s in total), and the radius denotes abundance (signal of FID detectors) in a logarithmic scale; MXT-5, MXT-1701—chromatographic columns. For all wine samples, AF was conducted by a *Saccharomyces cerevisiae* yeast strain (Essentiale Grand Cru); R3—a Rondo wine, spontaneous MLF (without LAB addition); R3 LAB—a Rondo wine, MLF induced by inoculation with LAB after AF; Z3—a Zweigelt wine, spontaneous MLF; Z3 LAB—a Zweigelt wine, MLF was carried out by LAB added after AF.

Fingerprints are shown in Figure 1 as two radar plots of the chromatograms obtained using the parallel columns/detectors of the ultra-fast GC system. The differences in the composition of selected wines evident in the chromatograms cannot be used as a basis for classification. Instead, data analysis techniques such as Principal Component Analysis (PCA) must be used.

**Figure 2:**
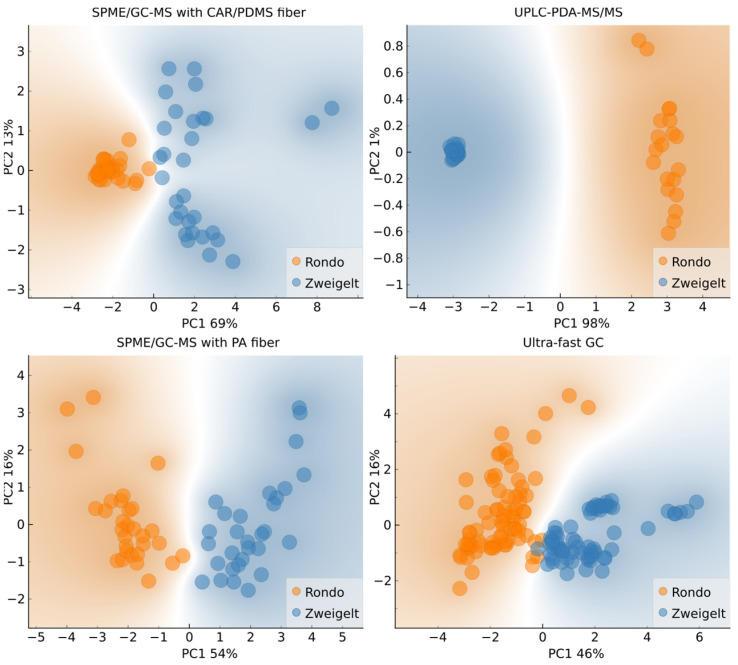
Biplots of the two first principal components with input variables selected based on grape variety variance; Rondo–Rondo wines in which AF was conducted by various yeast strains and MLF was spontaneous or induced (carried out without or with the addition of LAB, respectively); Zweigelt–Zweigelt wines in which AF was carried out using different yeast strains and MLF was spontaneous or induced by inoculation with LAB.

Based on the subplots in Figure 2, it can be seen that the samples of wine made from different grape varieties are mostly separated along the first principal component, although in the case of SPME/GC-MS with CAR/PDMS fiber, SPME/GC-MS with PA fiber, and ultra-fast GC, projecting the objects (number of samples, i.e., 20, multiplied by the number of replicates) to the PC1 axis would result in some overlap. Nonetheless, this is a good indication that the results of all four chromatographic methods produce outputs suitable for fingerprint-based machine-learning (ML) classification. Further, in the case of UPLC-PDA-MS/MS, there is a clear separation of features (analytes) along PC1, suggesting that the straightforward, classification tree-based approach might yield satisfactory results.

**Figure 3:**
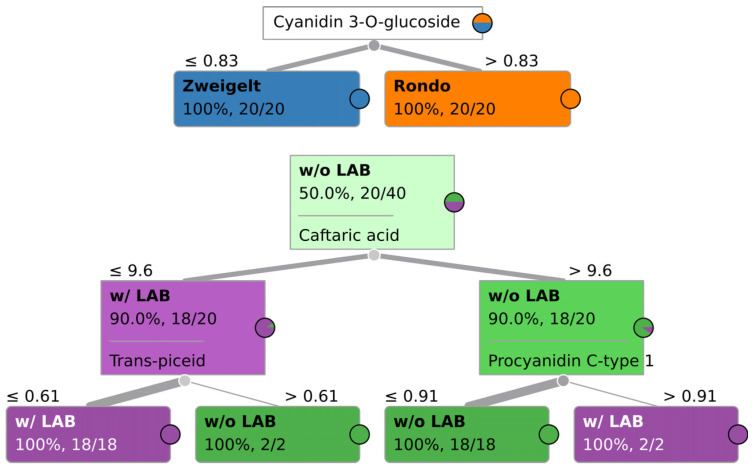
An example of classification tree, specific for this particular dataset, for determining grape variety and addition of LAB in the winemaking process based on the results of sample analysis using UPLC-PDA-MS/MS; Rondo–Rondo wines in which AF was carried out by various yeast strains and MLF was spontaneous or induced by inoculation with LAB; Zweigelt–Zweigelt wines in which AF was carried out by different yeast strains and MLF was spontaneous or induced by inoculation with LAB; w/o LAB–Rondo and Zweigelt wines in which AF was conducted by various yeast strains and MLF was carried out without the addition of LAB; w/LAB–Rondo and Zweigelt wines in which AF was conducted by various yeast strains and MLF was induced by inoculation with LAB.

Nine features that were most relevant in the classification of wines by grape variety for the three methods are presented in Supplementary Table S2. When examining the results of ML validation shown in Table 1, one can see that both ML classifiers performed very well, with classification accuracies of at least 98%, and yet with no obvious overfitting. It can be concluded that all four chromatographic methods produce results that are suitable for robust fingerprinting-based Quality Assurance/Quality Control (QA/QC) procedures when it comes to assuring that the wine was manufactured using a given grape variety. As shown in Figure 3, UPLC-PDA-MS/MS additionally allows one to determine whether a given wine was manufactured using Zweigelt or Rondo grapes based solely on the concentration of selected characteristic compounds, such as cyanidin-3-O-glucoside, through rule-based classification. The concentration of cyanidin-3-O-glucoside in wines produced from the Rondo variety was higher than in Zweigelt wines. It should be noted that this is not a universally applicable result and is only specific for this particular dataset and classification task. A more robust multivariate statistical analysis approach needs to be taken for more general applicability, or else a similar tree based on different criterion needs to be tailored to each case/classification task. Still, other authors [10,11,12,15] and the present authors in previous studies [13,14] obtained similarly satisfactory classifications of wines by grape variety based on the results of analyses of phenolic and volatile compounds determined using chromatographic methods.

**Figure 4:**
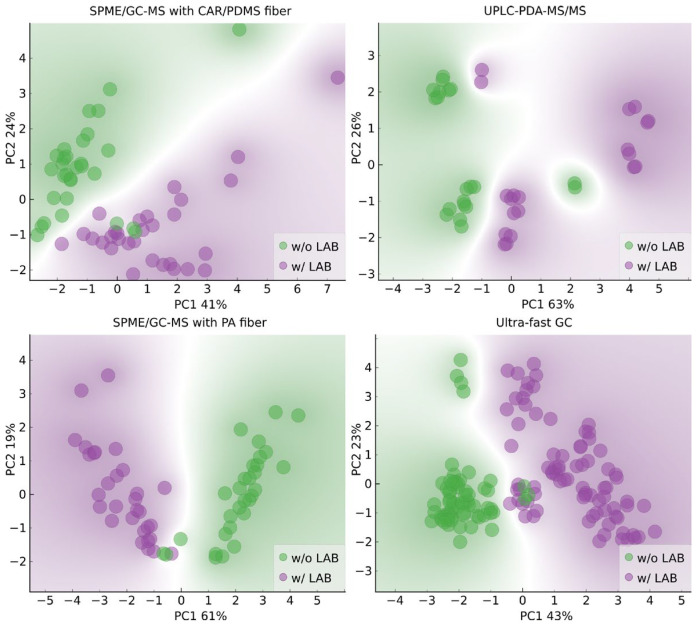
Biplots of the first two principal components with input variables selected based on variance with regard to whether LAB were added in the winemaking process; w/o LAB–Rondo and Zweigelt wines in which AF was conducted by various yeast strains and MLF was carried out without LAB addition; w/LAB–Rondo and Zweigelt wines in which AF was carried out by various yeast strains and MLF was induced by inoculation with LAB.

The literature data show that wines produced through spontaneous and induced MLF differ in the contents of phenolic and volatile compounds [2,3,26,27,28,29]. Therefore, we decided to verify whether it was possible to classify wines by MLF type based on the results obtained using chromatographic methods. Biplots of the PCA results with annotations based on whether LAB were added in the winemaking process are shown in Figure 4. The PCA results indicate that the classification task is less straightforward in this case. The outliers in Figure 4 are replicates of the same sample, Z3 (wine of the Zweigelt variety, in which AF was carried out by a *Saccharomyces cerevisiae* strain, Essentiale Grand Cru), and MLF was spontaneous.

Nine features that were most relevant in the classification of wines by the type of MLF for the three methods are presented in Supplementary Table S3. As was the case with the grape-variety-based classification, both Support Vector Machines (SVM) and Random Forest (RF) algorithms performed well, with a classification accuracy of no less than 91%, and greater than 98% in the case of ultra-fast GC (see Table 2). As expected, the rule-based classification approach for UPLC-PDA-MS/MS underperformed the ML-based classification algorithms, with an accuracy of 81% (see Figure 3). The receiver–operating curves for all three used models are shown in Supplementary Figure S1.

In general, it can be concluded that all four chromatographic methods produce results that can be reliably used as inputs for robust ML-based classifiers. Thus, the choice of a method for routine QA/QC analyses aimed at ascertaining wine parameters such as grape variety and use of LAB in the winemaking process should be based primarily on the practicality and greenness of each particular analytical approach.

**Figure 5:**
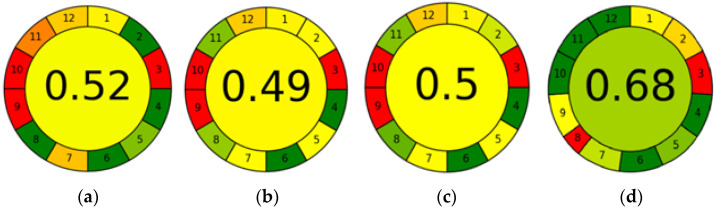
Results of AGREE analysis for UPLC-PDA-MS/MS (**a**), SPME/GC-MS with CAR/PDMS (**b**), SPME/GC-MS with PA fiber (**c**), and ultra-fast GC (**d**).

The results of the greenness assessment of the four chromatographic methods performed using the AGREE application are shown in Figure 5. In three of the methods, UPLC-PDA-MS/MS, SPME/GC-MS with CAR/PDMS fiber, and SPME/GC-MS with PA fiber, equal default weights were assigned to all the 12 principles (p 1–p 12), thus assuming that all the evaluation principles were equally important. However, in ultra-fast GC, a weight of 1 was assigned to principle 8 (number of analyses determined in a single run), because this method can be used to determine volatile compound profiles (fingerprints) but not volatile components.

In UPLC-PDA-MS/MS, the sampling procedure is conducted off-line (p 1), and 0.005 mL of the sample is needed (p 2). The measurement is performed in an off-line mode (p 3). The method does not involve sample preparation (p 4 and p 5). The method is semi-automatic (p 5). No derivatization agents are involved in the analysis (p 6). The total amount of waste is 10.35 g and mL combined, consisting of a syringe filter and UPLC mobile phase (p 7). Fifty-five analytes are determined in a single run, and three samples are analyzed per hour (p 8). UPLC-PDA-MS/MS is the most energy-intensive analytical technique (p 9). None of the reagents originate from bio-based sources (p 10), and the volume of toxic reagents is 6.65 mL (p 11). Acetonitrile is highly flammable and toxic to aquatic life, and formic acid is toxic to aquatic life as well as being corrosive (p 12).

SPME/GC-MS with CAR/PDMS fiber and SPME/GC-MS with PA fiber differ in the type of fiber used for microextraction and the values for principles 2 and 8. In both methods, the sampling procedure is performed off-line (p 1). In SPME/GC-MS with CAR/PDMS fiber, the volume of the sample required for analysis is 3 mL, whereas in SPME/GC-MS with PA fiber, it is 1.5 mL, because the sample is diluted two-fold (p 2). In both methods, the analytical device is positioned off-line (p 3). The sample preparation procedure consists of only one step—microextraction (p 4). The methods are manual but involve miniaturized sample preparation (p 5). The methods do not require the use of derivatizing agents (p 6). Analytical wastes include the sample itself (3 mL), NaCl (0.9 g), HCl (0.05 mL), 4-hydroxy-4-methyl-2-pentanone (0.1 mL), and septum (0.3 g) (p 7). The number of analytes determined by SPME/GC-MS is 46 when CAR/PDMS fiber is used and 67 when PA fiber is used; a 0.5 sample is analyzed per hour in both methods (p 8). The most energy-demanding device is the GC-MS (p 9). No reagents originate from bio-based sources (p 10). The methods require 0.15 mL of toxic solvents (p 11). HCl is toxic to aquatic life and corrosive, and 4-hydroxy-4-methyl-2-pentanone is toxic to aquatic life and highly flammable (p 12).

In ultra-fast GC, the sampling procedure is performed off-line (p 1). The volume of sample is 5 mL (p 2). The measurement is made off-line (p 3). The sample preparation procedure involves only one step—incubation and stirring (p 4). The method is semi-automatic and involves miniaturized sample preparation (p 5). The method does not require the use of derivatizing agents (p 6). The amount of waste generated is 1.8 g of cap with membrane (p 7). The analysis is not qualitative or quantitative, but it yields profiles of volatile compounds (fingerprints), and the sample throughput is ∼2.5 samples h^−1^ (p 8). The most energy demanding technique is GC (p 9). No reagents are used in this method (p 10, p 11, and p 12).

The final AGREE score for a given method is in the range of 0–1. The higher the score, the greener the method. With a score of 0.68, ultra-fast GC is the greenest of all the methods tested. This is because it generates the lowest amount of waste (p 7), consumes relatively little energy (p 9), and uses no reagents (p 10, p 11, and p 12). The weakness of this method is that it is not well-suited for the qualitative analysis of complex matrices (p 8). The other three methods are much less green and all have similar AGREE scores. UPLC-PDA-MS/MS obtained a score of 0.52. Even though it requires the lowest volume of sample (p 2) and has the highest analytical throughput (p 8), it generates the largest amount of waste, mainly solvents (p 7), consumes a lot of energy (p 9), uses reagents none of which come from bio-based sources (p 10), and also uses the highest volume of toxic reagents (p 11). SPME/GC-MS with CAR/PDMS fiber and SPME/GC-MS with PA fiber have scores of 0.49 and 0.5, respectively. The latter method achieved a slightly higher result than the first one because it requires a lower sample volume (p 2) and can be used to determine more analytes (p 8). The weaknesses of these methods are that they are manual (p 5), consume a lot of energy (p 9), and do not use reagents from bio-based sources (p 10).

**Figure 6:**
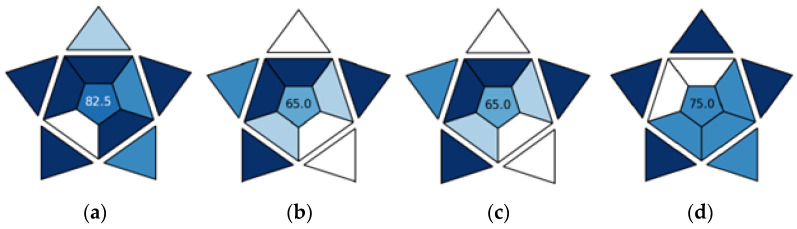
Results of BAGI analysis for UPLC-PDA-MS/MS (**a**), SPME/GC-MS with CAR/PDMS (**b**), SPME/GC-MS with PA fiber (**c**), and ultra-fast GC (**d**).

The results of the BAGI practicality evaluation of the four chromatographic methods are presented in Figure 6 and Supplementary Table S1. For three methods: UPLC-PDA-MS/MS, SPME/GC-MS with CAR/PDMS fiber, and SPME/GC-MS with PA fiber, one of four options was selected for each of the 10 attributes (a 1–a 10). However, for ultra-fast GC, no options were selected for attribute 1 (type of analysis) and attribute 2 (multi- or single-element analysis) because this method only yields fingerprints; for attribute 7 (reagents and materials), the best option (common commercially available reagents) was selected because this method does not use reagents.

The final BAGI score ranges from 25 to 100. The higher the score, the more practical a method is. UPLC-PDA-MS/MS is the most practical of all the methods considered with a score of 82.5, ultra-fast GC is less practical with a score of 75, and the least practical methods are SPME/GC-MS with CAR/PDMS fiber and SPME/GC-MS with PA fiber with a score of 65 each. The strong points of UPLC-PDA-MS/MS are the following: quantitative and confirmatory type of analysis (a 1), multi-element analysis of 55 compounds (a 2), assumption that 96 samples can be prepared simultaneously (a 4), the use of common commercially available reagents—acetonitrile and formic acid (a 6), no preconcentration in order to meet the required sensitivity (a 8), and a sample amount of 0.005 mL (a 10). The weak point of the method is the instrumentation that is not commonly available in most labs (a 3). The advantages of ultra-fast GC include simple instrumentation found in most labs (a 3), the highest sample throughput of 18 per h (a 6), no use of reagents (a 7), no preconcentration in order to meet the required sensitivity (a 8), and a sample amount of 5 mL (a 10). The disadvantage is that it does not allow for the performance of qualitative, quantitative, screening, or quantitative and confirmatory analysis, but instead yields fingerprints of volatile compounds (a 1 and a 2). Strong points of SPME/GC-MS with either CAR/PDMS or PA fiber are the quantitative and confirmatory type of analysis (a 1), the multi-element analysis of 46 and 67 compounds, respectively (a 2), no preconcentration in order to meet the required sensitivity (a 8), and sample amounts of 3 mL ad 1.5 mL, respectively (a 10). The weak points of the method are as follows: the assumption that only one sample can be prepared at a time (a 4), the use of commercially available reagents—SPME fibers—that are not typically stocked in QC labs (a 6), and manual treatment and analysis (a 9).

## 3. Materials and Methods

**Figure 7:**
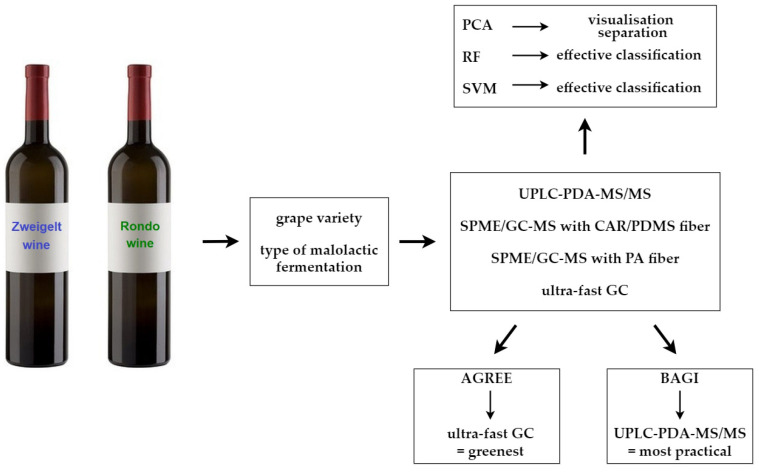
Materials and methods used in this paper.

The materials and methods are presented in Figure 7.

### 3.1. Winemaking Process and Wine Samples

The winemaking process was described in detail in our previous articles [13,14,25,30]. Briefly, the grapes of Zweigelt and Rondo varieties were harvested manually in 2017 in “Małe Dobre” and “Dom Bliskowice” vineyards, respectively. The vineyards are located in the Lublin Province, in Poland. AF was performed using five commercial yeast strains, either *S. cerevisiae* or *S. cerevisiae* × *S. bayanus*, for both Zweigelt and Rondo varieties. One part of the wines was subjected to spontaneous MLF without the addition of LAB, and the other part was produced by induced MLF with the addition of *O. oeni*. The experiments were performed in duplicate.

### 3.2. Ultra-Fast GC

The ultra-fast GC procedure was described in our previous publication [17]. Briefly, 5 mL of each wine was poured into 20 mL glass headspace vials, sealed with caps lined with a silicon–PTFE membrane, and incubated at 40 °C for 10 min. Wines were stirred at 500 rpm during the incubation. Static headspace analysis was performed using a Heracles II ultra-fast gas chromatography device with an HS100 autosampler (Alpha M.O.S., Toulouse, France). The device was fitted with two parallel 10 m columns packed with MXT-5 and MXT-1701 stationary phases (Restek, Bellefonte, PA, USA), respectively. Each column was coupled to a flame-ionization detector (μFID). Hydrogen of 6 N purity delivered using a Precision Hydrogen Trace 250 generator (Peak Scientific Instruments, Inchinnan, UK) was used as carrier gas. The static headspace sampling volume was 2.5 mL at 0.25 mL/s. The injector temperature was set to 200 °C, and the injection time was 15 s. During this time, the analytes were trapped on Tenax^®^ TA sorptive material at 40 °C, held for 20 s, and then purged into chromatographic columns through thermal desorption at 240 °C. The oven was ramped from 70 to 270 °C at 2 °C/s, and the acquisition duration was set to 100 s.

### 3.3. Statistical Analysis

The results of chromatographic analyses of wine samples using different methods served as inputs for ML analysis and were treated as fingerprints for classification based on grape variety and whether or not LAB were added in the winemaking process. The entire dataset consisted of 60 objects (individual analyses) and 46 features (i.e., separated compounds) for SPME/GC-MS with CAR/PDMS fiber, 40 objects and 55 features for UPLC-PDA-MS/MS, 60 objects and 67 features for SPME/GC-MS with PA fiber, and 147 objects and 133 features for ultra-fast GC (Supplementary Scheme S1). First, features with more than 50% missing values (e.g., due to the underlying concentrations of the separated compounds in the samples being below the LOQ) were purged from the dataset. Then, the features from each of the methods were normalized to the [0, 1] interval. Two target categorical features, namely grape variety and addition of LAB, were assigned to each object. PCA was performed in order to visualize the object distribution based on the two categories and to identify potential outliers.

To increase the ratio of objects to features for robust ML training, the dimensionality of the datasets was reduced to 9 using the Relief algorithm (Supplementary Tables S2 and S3), which is particularly useful for tasks involving classification into discrete categories [31,32]. This step was carried out separately for classification based on grape variety and LAB addition, with different features being selected for each task based on their correlation with the discrete target variable.

Finally, the applicability of two robust ML classifiers, namely, both RF and SVM [33,34,35], for predicting both grape variety and LAB addition based on the fingerprinting approach was assessed through validation. In the case of the RF classifier, the number of trees was set to 10, and the limit for subset splitting was set to 5. The SVM classifier featured the RBF kernel and was assigned a cost of 1.0 and a regression loss (ε) of 0.1. The validation involved stratified random sampling repeated 10 times, with 66% of the data assigned at random to the training set, and the remaining 34% to the testing set. The efficacy of the classifiers was assessed based on the area under the ROC curve, classification accuracy, and precision. Additionally, in the case of UPLC-PDA-MS/MS, which produced the most features, a more straightforward, rule-based approach to classification was also taken, involving a tree classifier (a precursor to RF), with data split into nodes based on class purity [36].

A multivariate statistical analysis was performed using the Scikit Learn 1.3.1 and Orange 3.35 Python packages [36,37].

### 3.4. Greenness and Blueness Measurements of Chromatographic Methods

The greenness and blueness of the four chromatographic methods that can be used for the varietal classification of wines and assessing the type of MLF were estimated using the AGREE application [23] and BAGI [20], respectively. The following chromatographic methods were compared, including three methods described in our previous publications: UPLC-PDA-MS/MS [15], SPME/GC-MS with CAR/PDMS fiber [25], SPME/GC-MS with PA fiber [13], and ultra-fast GC (used in this work).

## 4. Conclusions

It can be concluded that all four chromatographic methods are effective with regard to the varietal classification of wines and assessing the type of MLF. Nine analytes (features) were most relevant in both classification tasks for the three methods: UPLC-PDA-MS/MS, SPME/GC-MS with CAR/PDMS, and SPME/GC-MS with PA fiber (ultra-fast GC creates profiles of volatile compounds). RF and SVM had accuracies of at least 99% in varietal classification for four methods. The classification accuracy was not less than 91% with regard to the type of MLF.

The AGREE and BAGI findings for the four chromatographic methods show that ultra-fast GC is the most suitable approach for routine variety classification and MLF-type assessment. This method is the greenest because it generates the lowest amount of waste, consumes relatively little energy, and uses no reagents. It is the second most practical approach as it uses simple instrumentation available in most labs and provides the highest sample throughput per h. Its weakness is that the analysis is not qualitative, quantitative, screening, or quantitative and confirmatory, but instead fingerprints of volatile compounds are obtained. Although UPLC-PDA-MS/MS is generally the most practical method, it has the drawback of requiring instrumentation that is not commonly available in most labs. UPLC-PDA-MS/MS is much less green than ultra-fast GC. SPME/GC-MS with CAR/PDMS fiber and SPME/GC-MS with PA fiber are the least green and practical, primarily because they are manual, allow for the preparation of only one sample at a time, and use commercially available reagents that are not typically stocked in QC labs.

A methodology for rapidly assessing type of MLF was developed for the first time. The chromatographic methods discussed can be used to evaluate the MLF type with a high accuracy.

## Figures and Tables

**Table 1:** Performance statistics, i.e., the area under the receiver-operating curve (AUC), classification accuracy (CA), and precision of classification using 3 different ML approaches of different wine samples based on the grape variety.

Method	Model	AUC	CA	Precision
SPME/GC-MS with CAR/PDMS fiber	SVM	1.000	1.000	1.000
	RF	0.999	0.981	0.981
UPLC-PDA-MS/MS	SVM	1.000	0.993	0.993
	RF	1.000	0.993	0.993
	Tree	0.943	0.943	0.949
SPME/GC-MS with PA fiber	SVM	1.000	1.000	1.000
	RF	1.000	0.990	0.991
Ultra-fast GC	SVM	1.000	1.000	1.000
	RF	1.000	0.998	0.998

**Table 2:** Performance statistics, i.e., the area under the receiver–operating curve (AUC), classification accuracy (CA), and precision of the classification using 3 different ML approaches of different wine samples based on whether lactic acid bacteria were added in the winemaking process.

Method	Model	AUC	CA	Precision
SPME/GC-MS with CAR/PDMS fiber	SVM	0.976	0.910	0.910
	RF	0.954	0.933	0.934
UPLC-PDA-MS/MS	SVM	0.995	0.971	0.971
	RF	0.976	0.929	0.929
	Tree	0.843	0.807	0.808
SPME/GC-MS with PA fiber	SVM	0.999	0.986	0.986
	RF	0.983	0.957	0.959
Ultra-fast GC	SVM	1.000	0.996	0.996
	RF	0.999	0.984	0.984

## Data Availability

The data presented in this study are available on request from the corresponding author.

## Supplementary Materials

The following supporting information can be downloaded at: https://www.mdpi.com/article/10.3390/molecules29194667/s1, Table S1: BAGI scores for UPLC-PDA-MS/MS, SPME/GC-MS with CAR/PDMS, SPME/GC-MS with PA fiber and ultra-fast GC; Scheme S1: Objects and features used as inputs in the multivariate statistical analysis; Figure S1: ROCs for each method and model in classification based on MLF. FP = FN = 500, target probability: 50%; Table S2: Features selected for classification based on grape variety using Relief; Table S3: Features selected for classification based on MLF using Relief.
