# Supplementary material for: The Use of Ultra-Fast Gas Chromatography for Fingerprinting-Based Classification of Zweigelt and Rondo Wines with Regard to Grape Variety and Type of Malolactic Fermentation Combined with Greenness and Practicality Assessment
Source: Molecules. 2024 Oct 1;29(19):4667. doi: 10.3390/molecules29194667 (PMC11477865; doi:10.3390/molecules29194667)
Supplement: Supplementary file 1 [file molecules-29-04667-s001.zip › molecules-3197736-supplementary.pdf]

The use of ultra-fast gas chromatography for fingerprinting-based classification of Zweigelt and Rondo wines with regard to grape variety and type of malolactic fermentation combined with greenness and practicality assessment

Anna Stój [1,*], Wojciech Wojnowski [2,3], Justyna Płotka-Wasylka [2], Tomasz Czernecki [1] and Ireneusz Kapusta [4]

[1] Department of Biotechnology, Microbiology and Human Nutrition, Faculty of Food Science and Biotechnology, University of Life Sciences in Lublin, 8 Skromna Street, 20-704 Lublin, Poland; anna.stoj@up.lublin.pl (A.S.); tomasz.czernecki@up.lublin.pl (T.C.)

[2] Department of Analytical Chemistry, Faculty of Chemistry, Gdańsk University of Technology, 11/12G Narutowicza Street, 80-233 Gdańsk, Poland; wojciech.wojnowski@pg.edu.pl (W.W.); juswasyl@pg.edu.pl (J.P.-W.)

[3] Department of Chemistry, University of Oslo, 0315, Oslo, Norway

[4] Department of Food Technology and Human Nutrition, College of Natural Sciences, University of Rzeszow, 4 Zelwerowicza Street, 35-601 Rzeszów, Poland; ikapusta@ur.edu.pl (I.K.)

* Correspondence: anna.stoj@up.lublin.pl; Tel. +48 81 462 33 80

Table S1. BAGI scores for UPLC-PDA-MS/MS, SPME/GC-MS with CAR/PDMS, SPME/GC-MS with PA fiber and ultra-fast GC.

Number of attribute	Attribute	Method			
		UPLC-PDA-MS/MS	SPME/GC-MS with CAR/PDMS	SPME/GC-MS with PA fiber	ultra-fast GC
1	Type of analysis	quantitative and confirmatory	quantitative and confirmatory	quantitative and confirmatory	no option selected (fingerprints are obtained)
2	Multi- or single-element analysis	multi-element analysis for >15 compounds (precisely 55)	multi-element analysis for >15 compounds (precisely 46)	multi-element analysis for >15 compounds (precisely 67)	no option selected (fingerprints are obtained)
3	Analytical technique	instrumentation that is not commonly available in most labs (precisely LC-MS/MS)	sophisticated instrumentation (precisely GC-MS)	sophisticated instrumentation (precisely GC-MS)	simple instrumentation available in most labs (precisely GC-FID)
4	Simultaneous sample preparation	>95 (precisely 96)	1	1	13-95 (precisely 64)
5	The sample preparation	simple low-cost sample preparation is required (filtration)	miniaturized extraction sample preparation (precisely SPME)	miniaturized extraction sample preparation (precisely SPME)	simple low-cost sample preparation is required (incubation, stirring)
6	Samples per h	2-4 (precisely 3)	≤1 (0.5)	≤1 (0.5)	>10 (precisely 18)
7	Reagents and materials	common commercially available reagents (precisely acetonitrile, formic acid)	commercially available reagents that are not common in QC labs (precisely SPME fibres)	commercially available reagents that are not common in QC labs (precisely SPME fibres)	the best option was selected: common commercially available reagents (the method does not use reagents)
8	Preconcentration	no preconcentration is required; required sensitivity and/or legislation criteria are directly met	no preconcentration is required; required sensitivity and/or legislation criteria are directly met	no preconcentration is required; required sensitivity and/or legislation criteria are directly met	no preconcentration is required; required sensitivity and/or legislation criteria are directly met
9	Degree of automation	semi-automated with common devices (precisely sample manager)	manual treatment and analysis.	manual treatment and analysis.	semi-automated with common devices (precisely autosampler)
10	Amount of sample	≤100 μL (or mg) bioanalytical samples ≤10 mL (or g) food/ environmental (precisely 0.005 mL)	≤100 μL (or mg) bioanalytical samples ≤10 mL (or g) food/ environmental (precisely 3 mL)	≤100 μL (or mg) bioanalytical samples ≤10 mL (or g) food/ environmental (precisely 1.5 mL)	≤100 μL (or mg) bioanalytical samples ≤10 mL (or g) food/ environmental (precisely 5 mL)
Score		82.5	65.0	65.0	75.0

Scheme S1. Objects and features used as inputs in the multivariate statistical analysis

Target variables:

- Grape [categorical]: {Rondo, Zweigelt}
- LAB [categorical]: {yes, no}

Meta attributes: {wine, sample}

Features [numeric] SPME/GC-MS with CAR/PDMS fiber:

{
Ethyl butanoate, Propanol-1-ol, Ethyl 3-methylbutanoate, 2-Methylpropan-1-ol , 3-Methylbutyl acetate , 4-Methyl-3-penten-2-one , Butan-1-ol, 3-Methylbutan-1-ol, Pentan-1-ol, Hexyl acetate, 3-Hydroxybutan-2-one, 4-Methylpentan-1-ol , 3-Methylpentan-1-ol, Ethyl heptanoate, Ethyl 2-hydroxypropanoate, Hexan-1-ol, 3-Ethoxypropan-1-ol, Methyl octanoate, (Z)-2-Hexen-1-ol , Ethyl octanoate, Octen-3-ol, Heptan-1-ol, Acetic acid , 2-Ethylhexan-1-ol , 3-Ethyl-4-methylpentan-1-ol, Benzaldehyde, Butane-2,3-diol, Propanoic acid, Octan-1-ol, 3-Methylbutyl 2-hydroxypropanoate, 2-Methylpropanoic acid, Propane-1,2-diol, 2-(2-Ethoxyethoxy)-ethanol, Ethyl 2-furoate, Dihydrofuran-2(3H)-one, Ethyl decanoate, 4-Methylbenzaldehyde, Diethyl butanedioate, 3-(Methylsulfanyl)propan-1-ol, Decan-1-ol, 2-Phenylethyl acetate , Hexanoic acid, Phenylmethanol, 2-Phenylethanol, Octanoic Acid, 3,5-Di-tert-butylphenol
}

Features [numeric] UPLC-PDA-MS/MS:

{
Trans-piceid, Cis-piceid, Trans-resveratrol, Cis-resveratrol, Delphinidin 3-O-glucoside-5-O-glucoside, Cyanidin 3-O-glucoside-5-O-glucoside, Delphinidin 3-O-glucoside, Petunidin 3-O-glucoside-5-O-glucoside, Peonidin 3-O-glucoside-5-O-glucoside, Malvidin 3-O-glucoside-5-O-glucoside, Cyanidin 3-O-glucoside, Petunidin 3-O-glucoside, Peonidin 3-O-glucoside, Malvidin 3-O-glucoside, Delphinidin 3-O-(6''-O-acetyl)-glucoside, Cyanidin 3-O-(6''-O-acetyl)-glucoside, Petunidin 3-O-(6''-O-acetyl)-glucoside, Petunidin 3-O-(6''-O-acetyl)-glucoside-5-O-glucoside, Delphinidin 3-O-(6''-O-coumaryl)-glucoside, Malvidin 3-O-(6''-O-acetyl)-glucoside, Malvidin 3-O-(6''-O-coumaryl)-glucoside-5-O-glucoside, Peonidin 3-O-(6''-O-coumaryl)-glucoside-5-O-glucoside, Peonidin 3-O-(6''-O-acetyl)-glucoside, Cyanidin 3-O-(6''-O-coumaryl)-glucoside, Petunidin 3-O-(6''-O-coumaryl)-glucoside, Delphinidin 3-O-(6''-caffeoyl)-glucoside, Peonidin 3-O-(6''-O-coumaryl)-glucoside, Malvidin 3-O-(6''-O-coumaryl)-glucoside, Myricetin-3-O-rutinoside, Myricetin-3-O-glucoside, Quercetin 3-O-glucuronide Isorhamnetin 3-O-glucoside, Quercetin 3-O-glucoside, Quercetin 3-O-rutinoside, Dihydroquercetin 3-O-ramnoside, Procyanidin B1, Procyanidin B-type 1, Procyanidin C1, (+) Catechin, Procyanidin C-type 1, Procyanidin B-type 2, Procyanidin B2, (-) Epicatechin, Procyanidin C-type 2, Procyanidin C-type 3, Procyanidin B-type 3, Procyanidin B-type 4, Gallic acid, Protocatechuic acid, Caftaric acid, Coutaric acid, Caffeic acid, Ferulic acid, p-Coumaric acid, Coumaric acid
}

Features [numeric] SPME/GC-MS with PA fiber:

{
Ethyl 3-methylbutanoate, 2-Methylpropan-1-ol, 4-Methyl-3-penten-2-one, Butan-1-ol, 3-Methylbutan-1-ol, Pentan-1-ol, 3-Hydroxybutan-2-one, 4-Methylpentan-1-ol, 3-Methylpentan-1-ol, Ethyl 2-hydroxypropanoate, Hexan-1-ol, (E)-3-Hexen-1-ol, 3-Ethoxypropan-1-ol, (Z)-3-Hexen-1-ol, Methyl octanoate, Ethyl octanoate, (Z)-Linalool oxide, Octen-3-ol, Heptan-1-ol, Acetic acid, 2-Methylpropyl 2-hydroxypropanoate, 2-Ethylhexan-1-ol, 3-Ethyl-4-methylpentan-1-ol, Benzaldehyde, Ethyl nonanoate, Butane-2,3-diol, 3,7-Dimethyl-1,6-octadien-3-ol β-Linalol, Propanoic acid, Octan-1-ol, 3-Methylbutyl 2-hydroxypropanoate, 2-Methylpropanoic acid, Propane-1,2-diol, 3,7-Dimethyl-1,5,7-octatrien-3-ol Hotrienol, 2-(2-Ethoxyethoxy)-ethanol, Ethyl 2-furoate, Dihydrofuran-2(3H)-one, Ethyl decanoate, 4-Methylbenzaldehyde, Nonan-1-ol, Ethyl benzoate, Diethyl butanedioate, Ethyl 9-decenoate, 3-Cyclohexene-1-methanol, alpha,alpha,4-trimethyl- (α-Terpineol), 3-(Methylsulfanyl)propan-1-ol, 1,1,6-Trimethyl-1,2-dihydronaphthalene (TDN), Decan-1-ol, Methyl 2-hydroxy benzoate, Ethyl phenylacetate, 2-Phenylethyl acetate, (E)-1-(2,6,6-trimethyl-1,3-cyclohexadien-1-yl)-2-buten-1-one β-Damascenone, Ethyl dodecanoate, Hexanoic acid, (E)-6,10-dimethyl-5,9-Undecadien-2-one (Geranylacetone), Phenylmethanol, Ethyl 3-phenylpropanoate, 2-Phenylethanol, Dodecan-1-ol, Diethyl-2-hydroxybutanedioate, Octanoic Acid, Nonanoic acid, Methyl hexadecanoate, Ethyl hexadecanoate, Decanoic acid, (E,E)-3,7,11-trimethyl-2,6,10-Dodecatrien-1-ol (E,E)-Farnesol, Hexadecan-1-ol, Benzoic acid, Dodecanoic acid
}

In the case of the Ultra-fast GC method the [numeric] features were based on partially-resolved peaks from both columns/detectors labeled with the nearest integer RT and not assigned to any compound.

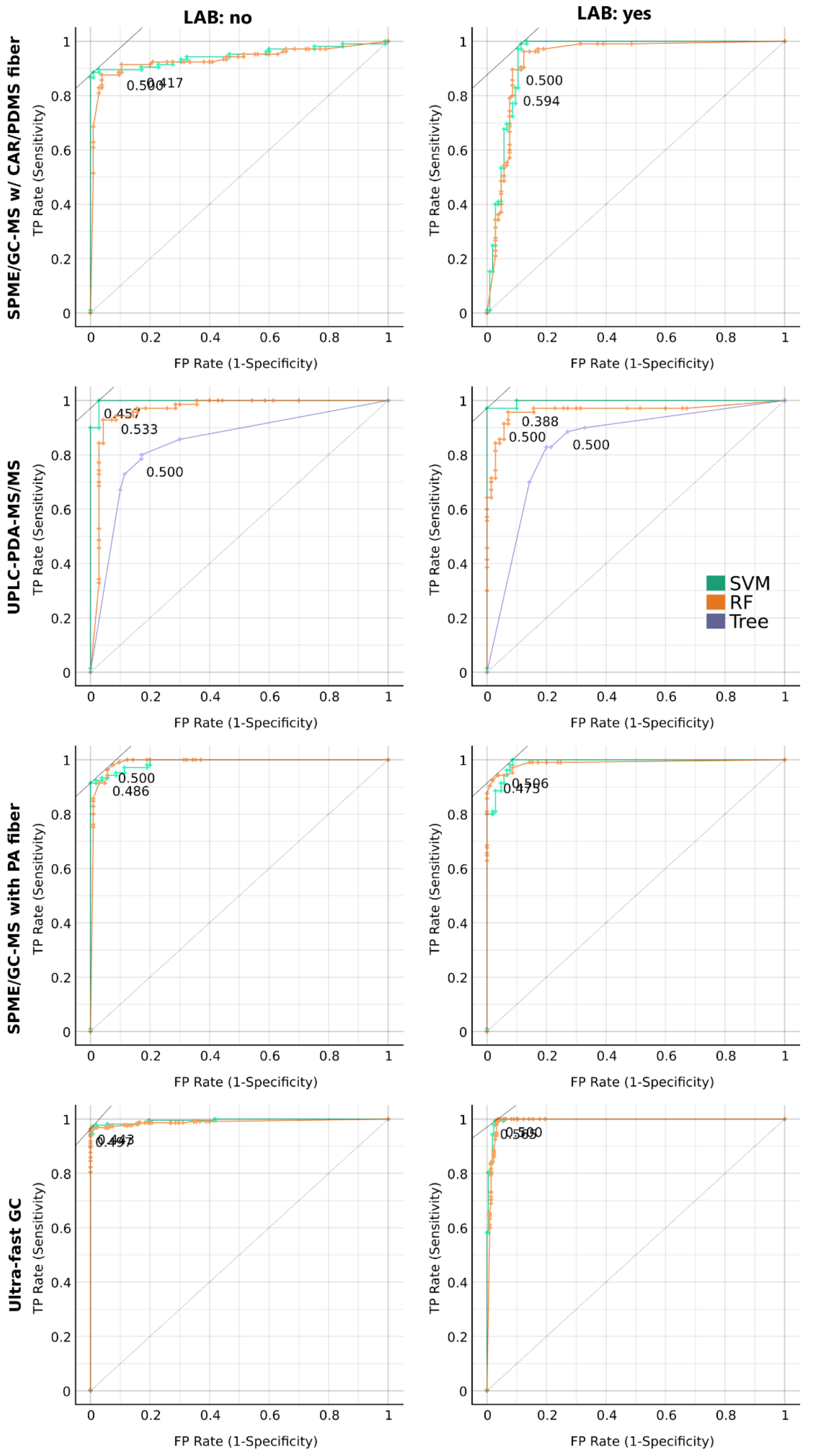

Figure S1. ROCs for each method and model in classification based on MLF. FP=FN=500, target probability: 50%.

Table S2. Features selected for classification based on grape variety using Relief algorithm

#	Feature	Relief score
SPME/GC-MS with CAR/PDMS fiber		
1	3-Ethyl-4-methylpentan-1-ol	0.269
2	4-Methylpentan-1-ol	0.174
3	Phenylmethanol	0.157
4	(Z)-2-Hexen-1-ol	0.155
5	4-Methylbenzaldehyde	0.14
6	Hexan-1-ol	0.128
7	Heptan-1-ol	0.125
8	Hexanoic acid	0.124
9	Diethyl butanedioate	0.118
UPLC-PDA-MS/MS		
1	Petunidin 3-O-(6''-O-acetyl)-glucoside-5-O-glucoside	0.92
2	Malvidin 3-O-(6''-O-coumaryl)-glucoside-5-O-glucoside	0.838
3	Delphinidin 3-O-glucoside-5-O-glucoside	0.836
4	Petunidin 3-O-glucoside-5-O-glucoside	0.831
5	Dihydroquercetin 3-O-ramnoside	0.816
6	Cyanidin 3-O-glucoside	0.815
7	Peonidin 3-O-glucoside-5-O-glucoside	0.811
8	Malvidin 3-O-glucoside-5-O-glucoside	0.794
9	Peonidin 3-O-(6''-O-coumaryl)-glucoside-5-O-glucoside	0.785
SPME/GC-MS with PA fiber		
1	3-Ethyl-4-methylpentan-1-ol	0.364
2	3,7-Dimethyl-1,5,7-octatrien-3-ol Hotrienol	0.327
3	Octen-3-ol	0.194
4	Hexanoic acid	0.189
5	Butane-2,3-diol	0.167
6	3-(Methylsulfanyl)propan-1-ol	0.163
7	Octanoic Acid	0.141
8	Acetic acid	0.135
9	Benzaldehyde	0.13

Table S3. Features selected for classification based on MLF using Relief algorithm

#	Feature	Relief score
SPME/GC-MS with CAR/PDMS fiber		
1	Ethyl 2-hydroxypropanoate	0.276
2	3-Methylbutyl 2-hydroxypropanoate	0.208
3	Acetic acid	0.152
4	Propanoic acid	0.102
5	Dihydrofuran-2(3H)-one	0.04
6	3-Methylbutyl acetate	0.039
7	Ethyl 2-furoate	0.036
8	Ethyl heptanoate	0.036
9	Phenylmethanol	0.02
UPLC-PDA-MS/MS		
1	Caffeic acid	0.373
2	Procyanidin C-type 1	0.248
3	Caftaric acid	0.221
4	Trans-piceid	0.221
5	Coutaric acid	0.184
6	Coumaric acid	0.179
7	Ferulic acid	0.136
8	Trans-resveratrol	0.128
9	p-Coumaric acid	0.123
SPME/GC-MS with PA fiber		
1	Ethyl 2-hydroxypropanoate	0.366
2	3-Methylbutyl 2-hydroxypropanoate	0.316
3	2-Methylpropyl 2-hydroxypropanoate	0.22
4	Acetic acid	0.19
5	Diethyl-2- hydroxybutanedioate	0.175
6	Ethyl 2-furoate	0.124
7	2-Phenylethyl acetate	0.115
8	Benzaldehyde	0.102
9	Methyl 2-hydroxy benzoate	0.078
